# Quantitative MRI in cardiometabolic disease: From conventional cardiac and liver tissue mapping techniques to multi-parametric approaches

**DOI:** 10.3389/fcvm.2022.991383

**Published:** 2023-01-23

**Authors:** Anastasia Fotaki, Carlos Velasco, Claudia Prieto, René M. Botnar

**Affiliations:** ^1^School of Biomedical Engineering and Imaging Sciences, King’s College London, London, United Kingdom; ^2^School of Engineering, Pontificia Universidad Católica de Chile, Santiago, Chile; ^3^Institute for Biological and Medical Engineering, Pontificia Universidad Católica de Chile, Santiago, Chile; ^4^Millennium Institute for Intelligent Healthcare Engineering, Santiago, Chile

**Keywords:** cardiometabolic disease, MRI, tissue characterization, mapping, multiparametric mapping

## Abstract

Cardiometabolic disease refers to the spectrum of chronic conditions that include diabetes, hypertension, atheromatosis, non-alcoholic fatty liver disease, and their long-term impact on cardiovascular health. Histological studies have confirmed several modifications at the tissue level in cardiometabolic disease. Recently, quantitative MR methods have enabled non-invasive myocardial and liver tissue characterization. MR relaxation mapping techniques such as T_1_, T_1ρ_, T_2_ and T_2_* provide a pixel-by-pixel representation of the corresponding tissue specific relaxation times, which have been shown to correlate with fibrosis, altered tissue perfusion, oedema and iron levels. Proton density fat fraction mapping approaches allow measurement of lipid tissue in the organ of interest. Several studies have demonstrated their utility as early diagnostic biomarkers and their potential to bear prognostic implications. Conventionally, the quantification of these parameters by MRI relies on the acquisition of sequential scans, encoding and mapping only one parameter per scan. However, this methodology is time inefficient and suffers from the confounding effects of the relaxation parameters in each single map, limiting wider clinical and research applications. To address these limitations, several novel approaches have been proposed that encode multiple tissue parameters simultaneously, providing co-registered multiparametric information of the tissues of interest. This review aims to describe the multi-faceted myocardial and hepatic tissue alterations in cardiometabolic disease and to motivate the application of relaxometry and proton-density cardiac and liver tissue mapping techniques. Current approaches in myocardial and liver tissue characterization as well as latest technical developments in multiparametric quantitative MRI are included. Limitations and challenges of these novel approaches, and recommendations to facilitate clinical validation are also discussed.

## 1. Introduction

Quantitative MRI (QMRI) measures physical tissue values, related to the nuclear spin of protons in water. It includes the T_1_-, T_2_-, T_2_*-, T_1ρ_ -relaxation times and the proton density. The respective parameter maps provide quantitative parameter values for each voxel, which carry information about the corresponding structural environment of the protons. QMRI can be used to assess microstructural alterations related to tissue remodeling and has emerged as valuable imaging modality for myocardial and hepatic tissue characterization (1, 2). QMRI has been incorporated in standardized diagnostic clinical protocols in various pathologies, including inflammatory cardiomyopathies (3), amyloidosis (4), Anderson-Fabry disease (5) and iron overload (1, 2, 6). It has also been proposed by both the European Association for the Study of the Liver and the American Association for the Study of Liver Disease as a non-invasive diagnostic tool for tissue characterization in Non-alcoholic Fatty Liver Disease (NAFLD) (7, 8). QMRI facilitates direct quantitative comparison of tissue maps in the same individual with chronic disease over time and allows more accurate longitudinal monitoring of the disease, thereby enabling an individualized characterization and more objective patient assessment.

Cardiometabolic disease, which describes a clustering of disorders that touch upon the interface between cardiovascular disease (hypertension, atherosclerosis) and metabolic disease states (insulin resistance, diabetes, adiposity, NAFLD) (9), is a chronic disease state and a major cause of morbidity worldwide. The reported prevalence is 33–35% in adults and is associated with an increased risk of adverse cardiovascular events and all-cause mortality (10, 11). Cardiometabolic disease is challenging for physicians to manage because it can be present for years before becoming clinically apparent. Histological and functional alterations have been observed in the heart and liver, in addition to the skeletal muscle, liver, pancreas, adipose tissue and microcirculation (12). Numerous studies suggest that QMRI may add valuable information by identifying microstructural tissue damage early in the disease process, allowing for instituting and maintaining optimum health behaviors and treatment strategies, at a time when it is likely to be most effective.

The objective of this review is to provide an overview of parametric QMRI in cardiac and hepatic tissue characterization in cardiometabolic disease. First, we describe cardiac and hepatic tissue structural changes that occur in the primary manifestations of cardiometabolic disease, namely in diabetes, hypertension and atherosclerosis, as a framework for understanding how QMRI can be utilized to assess these changes. Then, we describe single-parameter mapping techniques and their clinical applications in the corresponding disease states. Lastly, we describe emerging multiparametric approaches in heart and liver, which are promising for comprehensive understanding of this multi-faceted disease.

## 2. Microscopic tissue alterations in cardiometabolic disease

### 2.1. Diabetic cardiomyopathy

**FIGURE 1:**
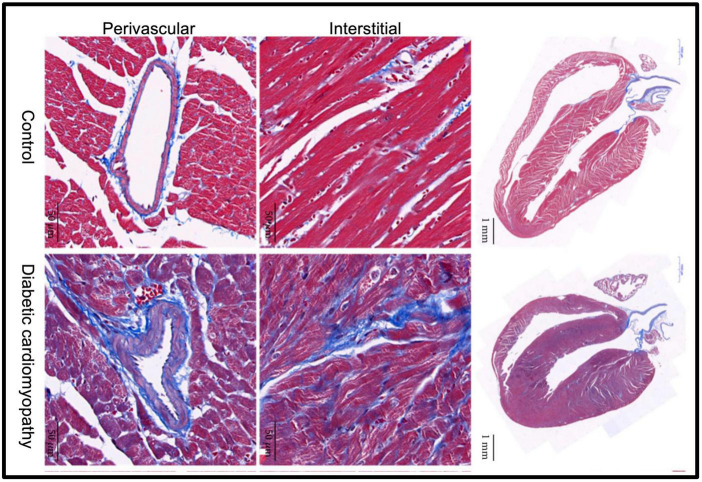
Fibrosis plays a crucial role in the development of diabetic cardiomyopathy. Representative images of Masson’s trichrome staining of a longitudinal section of the heart of control and diabetic mice (scale bar 1 mm). Magnified views show extracellular collagen deposition in the interstitial (scale bar 50 μm) and perivascular (scale bar 50 μm) space. Compared to the control group, diabetic cardiomyopathy mouse hearts displayed markedly increased collagen content both in the interstitial and perivascular space. Adapted from Li et al. with permission (24).

Type 2 diabetes mellitus (T2DM) is estimated to affect 6% of the world’s population (13) and is considered a coronary heart disease risk equivalent (14–17). The pathogenesis of the cardiac morbidity is multifactorial (18, 19). It has been proposed that metabolic modifications induced by hyperglycaemia, insulin resistance and hyperlipidaemia cause an aberrant use of fatty acids for energy generation (20). Fatty acid may saturate ß-oxidation and accumulate in the cytosol, leading to lipotoxic effects. Furthermore, hyperglycemia elicits reactive oxygen species and advanced glycation product formation, which lead to cardiac glucotoxicity. Both, the lack of fuel and lipo/gluco-toxicity as well as disturbances in mitochondrial energetics are triggering cardiac low-grade chronic inflammation, fibrosis and contractile dysfunction (21). Histological studies have confirmed corresponding changes in the myocardium of diabetic patients and animals, including the presence of diffuse myocardial and perivascular fibrosis (22–24; Figure 1), increased quantities of matrix collagen, inflammation, myocyte hypertrophy, myocardial steatosis and increased apoptosis (25–29). These pathophysiological changes often evolve quiescently to heart failure; and the prevalence of heart failure in T2DM is ranging from 19 to 26% (30, 31). It is therefore of clinical relevance to comprehend early alterations of cardiac tissue composition in T2DM and the progress from subclinical disease to more advanced disease stage manifesting clinically.

### 2.2. Hypertensive cardiomyopathy

**FIGURE 2:**
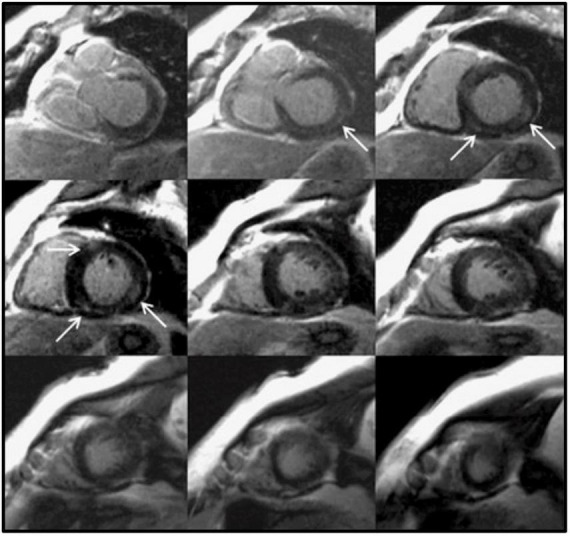
Focal myocardial fibrosis in a hypertensive patient. Late gadolinium enhancement in a 63-year-old female patient with longstanding hypertension. The arrows show an area of intramyocardial late gadolinium enhancement in the basal and mid inferoseptal and inferolateral segments. This is not the most typical fibrosis pattern in hypertension, which is usually diffuse. Adapted from Maceira et al. with permission (36).

Arterial hypertension is part of the constellation of disorders that constitute the cardiometabolic disease and is associated with an estimated 54% of strokes and 47% of ischemic heart disease worldwide (32, 33). The pathogenesis of hypertensive heart disease involves primarily cardiomyocyte hypertrophy, providing adaptive response to pressure overload (involving effects of growth factors, cytokines and neurohormones, and genetic predisposition) (34, 35). The alterations in the cellular and non-cellular (extracellular matrix) level induce structural remodeling of the myocardium with fibrosis of the muscle and perivascular space, medial hypertrophy of intramyocardial coronary vasculature, microangiopathy with decreased coronary reserve and development of epicardial coronary stenoses (36, 37). Myocardial fibrosis has been documented histologically in hypertensive hearts in subjects with hypertension (HTN) and left ventricular hypertrophy (LVH) (38). Myocardial fibrosis can be focal, referred to as replacement fibrosis, or diffuse, also known as interstitial fibrosis and is the most typical pattern in hypertensive heart disease (36) (Figure 2). Myocardial fibrosis predisposes patients to diastolic and systolic dysfunction, myocardial ischemia, and arrhythmias (39). It has been also demonstrated that treatment with inhibitors of angiotensin converting enzyme reduces collagen content and left ventricular stiffness with potential improvement in diastolic and systolic function, and perhaps outcomes (40). Thus, monitoring myocardial tissue alterations in hypertensive patients could enable risk stratification, inform treatment strategies, and monitor response.

### 2.3. Atherosclerotic cardiovascular disease

Atherosclerotic Cardiovascular Disease constitutes an important aspect of cardiometabolic syndrome and remains a leading cause of morbidity and mortality worldwide (41). The atherogenic process is primarily an inflammatory process and consists of several cellular and molecular interactions, including fatty tissue accumulation, platelet aggregation, abnormal vasomotor function, and can potentially culminate in atherosclerotic plaque formation, erosion, rupture or concomitant thrombus formation (42, 43), impeding blood flow and leading to tissue ischemia. In an acute ischemic event, the infarcted myocardial regions undergo a complex process of invasion, transformation and apoptosis of various cell types, including inflammatory cells and myofibrolasts, before remodeling to fibrotic scar tissue. Occasionally lipomatous metaplasia of the scar tissue ensues (44, 45). Fibrosis has also been histologically observed in non-infarcted regions of the heart as a result of left ventricular remodeling in patients with severe coronary atherosclerosis (46, 47). It is hypothesized that coronary artery stenosis, induced by atherosclerosis, impairs perfusion and causes chronic hypoxia with myocyte loss with consequent “reparative” collagen synthesis, contributing to interstitial collagen accumulation (48). Furthermore, there is ample evidence supporting the association of inflammation with the initiation and progression of atherosclerosis (43, 49). Atherectomy specimens have demonstrated the migration of the inflammatory cells in the arterial endothelium and that the inflammatory burden contributes to atherogenesis and adverse events (43).

### 2.4. Non-alcoholic fatty liver disease

NAFLD is considered the hepatic manifestation of the metabolic syndrome and constitutes one of the most common causes of chronic liver disease, with an estimated worldwide prevalence of around 25% (50). It is characterized by excessive fat accumulation in the hepatic tissue that is not attributable to consumption of alcohol (8). This condition may range histologically from simple non-alcoholic fatty liver, which is considered a benign condition, to non-alcoholic steatohepatitis (NASH), which additionally involves various stages of inflammation to tissue necrosis (8). Evidence from several studies suggests that all-cause mortality and more specifically cardiovascular-related mortality is higher in patients with NASH, and this is independent of the risk conferred by traditional risk factors and components of the metabolic syndrome (51–53). There is therefore a clinical need for reliable non−invasive biomarkers at the tissue level for the assessment of NAFLD and NASH (54).

## 3. Single-parameter mapping techniques in cardiometabolic disease

Parametric mapping requires the acquisition of a series of weighted images with different contrasts. These contrasts are generated by varying timing parameters such as echo times or inversion times. Fitting the series of weighted images to the corresponding signal model, in a pixel-wise manner, enables the generation of a quantitative map of the tissue relaxation, expressed in units of time (e.g., milliseconds). Single-parameter mapping techniques include T_1_ mapping, T_2_ mapping, T_2_* mapping and T1rho mapping. Extracellular volume can be generated from native (pre-contrast) and post contrast T_1_ mapping. Proton density fat fraction (PDFF) is a ratio, expressed as a percentage, of the fraction of the MRI-visible protons attributable to fat divided by all MRI-visible protons in that region of the liver attributable to fat and water. A brief introduction to each of these maps and their application in cardiometabolic disease is given below. The latter is also summarized in Table 1. The reader is referred to (55, 56) for further reading about the specific mapping techniques.

**TABLE 1:** *In vivo* CMR studies with conventional single-parameter mapping techniques in patients with cardiometabolic disease.

References	Study design	Patient characteristics	Reference standards	CMR methods	Accuracy/Correlation
Permutt et al. (100)	Prospective cross-sectional	51 NAFLD adults	51 corresponding biopsies	PDFF	PDFF correlated with histology-determined steatosis, (*r*2 = 0.54, *P* < 0.0001)
Wong et al. (87)	Cross-sectional prospective	231 T2DM adults	945 non-diabetic patients referred for CMR	ECV	ECV: 30% (26.9, 32.7) T2DM vs 28.1% (25.0, 31) HV, *P* < 0.001
Idilman et al. (166)	Retrospective observational	70 NAFLD adults	Corresponding liver biopsies	PDFF	PDFF correlated with biopsy-determined steatosis, (*r* = 0.86, *P* = 0.02) PDFF correlated less strongly with biopsy-determined steatosis when fibrosis was present, (*r* = 0.6 vs *r* = 0.859, respectively; *P* = 0.020) PDFF correlated better in mild hepatic steatosis than that of moderate or severe steatosis (*r* = 0.835 and *r* = 0.402, respectively; *P* = 0.003) PDFF measurement of 15.03% (area under the curve, 0.95; 95% confidence interval: 0.91, 1.00) differentiates moderate or severe steatosis from mild or no hepatic steatosis, with a sensitivity of 93.0% and a specificity of 85.0%, and respective positive and negative predicted values of 91.0% and 88.0%
Tang et al. (104)	Prospective cross-sectional	77 NAFLD adults	77 corresponding biopsies	PDFF	PDFF was significantly correlated with histologic steatosis grade (ρ = 0.69, *P* < 0.001). Area under the receiver operating characteristic curves was 0.989 (95% confidence interval: 0.968, 1.000) for distinguishing patients with steatosis grade 0 (*n* = 5) from those with grade 1 or higher (*n* = 72); 0.825 (95% confidence interval: 0.734, 0.915) to distinguish those with grade 1 or lower (*n* = 31) from those with grade 2 or higher (*n* = 46); 0.893 (95% confidence interval: 0.809, 0.977) to distinguish those with grade 2 or lower (*n* = 58) from those with grade 3 (*n* = 19).
Shah et al. (89)	Cross-sectional prospective	11 T2DM obese adolescents	10 non-T2DM obese adolescents 12 HV		ECV: 37.6% (33.6%, 40.7%) T2DM obese vs 32.8% (27.8%, 34.5%) non-T2DM obese, *P* = 0.03 ECV: 26.4% (25.3%, 27.1%) T2DM obese vs 37.6% (33.6%, 40.7%) HV, *P* = 0.03 ECV was associated with hemoglobin A1c (*r* = 0.76, *P* < 0.0001)
Banerjee et al. (110)	Comparative prospective	90 NAFLD/NASH adults	Histological specimens within 1 month 7 HV	Native T1 and T2* Estimated cT1	cT1 correlated with increasing liver fibrosis *r*_*s*_ = 0.68, 95% CI 0.54–0.78, *p* < 0.0001
Kuruvilla et al. (38)	Cross-sectional prospective	20 HTN LVH, 23 HTN non-LVH	22 HV	Native T1 ECV	Native T1: 996 ± 32.5 ms HTN LVH vs 967.4 ± 35 ms HV, *P* = 0.007 Native T1: 974.0 ± 33.6 ms HTN non-LVH vs 976.4 ± 35 ms HV, *P* = not statistically significant ECV: 29% ± 3 HTN LVH vs 26% ± 2 HV, *P* = 0.006 ECV: 27% ± 2 HTN non-LVH vs 26% ± 2 HV, *P* = 0.6
Treibel et al. (67)	Observational prospective	40 well-controlled HTN adults	50 HV		Native T1: 997 ± 27 ms HTN with LVH vs 948 ± 31 ms HTN no LVH, *p* < 0.001 Native T1: 955 ± 30 ms HTN versus 965 ± 38 ms HV, *p* = 0.16 ECV: 27.1% ± 2.7 HTN vs 26.1 ± 2.4, *P* = 0.06 ECV: 28.8 ± 2.8% HTN LVH vs. 26.2 ± 2.2 HTN no LVH, *p* < 0.01)
Doycheva et al. (102)	Prospective cross-sectional	100 T2DM adults	None	PDFF	PDFF, median (IQR): 12.3 (9.2) T2DM NAFLD vs 2.7 (1.9) T2DM no NAFLD, *P* < 0.0001
Levelt et al. (20)	Cross-sectional prospective	46 T2DM adults	20 HV	Native T1 ECV	Native T1: 1,194 ± 32 ms T2DM vs 1,184 ± 28 ms HV, *P* = 0.23 ECV: 29% ± 2 T2DM vs 29% ± 3 HV, *P* = 0.77
Rodrigues et al. (66)	Observational prospective	88 HTN (41 normal LV; 15 Conc-REMDL; Conc-LVH 24; Ecc LVH 8	29 HV	Native T1 ECV	Native T1: 1,031 ± 35 ms HTN normal LV vs 1,024 ± 41 ms HV, *p* = reported as not statistically significant Native T1: 1,029 ± 45 ms HTN Conc-REMDL vs 1,024 ± 41 ms HV, *p* = reported as not statistically significant Native T1: 1,054 ± 41 ms HTN Conc-LVH vs 1,024 ± 41 ms HV, *p* = 0.007 Native T1: 1,062 ± 41 ms HTN Ecc-LVH vs 1,024 ± 41 ms HV, *p* = 0.017 ECV: 29% ± 4 HTN Conc-LVH vs 27% ± 3 HTN normal LV, *p* < 0.0001 ECV: 29% ± 4 HTN Conc-LVH vs 26% ± 3 HTN Conc-REMDL, *P* = 0.012, *p* < 0.0001 ECV: 30% ± 3 HTN Ecc-LVH vs 27% ± 3 HTN normal LV, *P* = 0.6 ECV: 30% ± 3 HTN Ecc-LVH vs 26% ± 3 HTN Conc-REMDL, *P* = 0.021
Swoboda et al. (61)	Case-controlled observational	100 T2DM adults (50 ACR+ve T2DM, 50 ACR-ve T2DM)	30 HV	Native T1 ECV	Native T1: 1,232 ± 36 ms T2DM vs 1,210 ± 47 ms HV, *P* = 0.0.02 Native T1: 1,253 ± 66 ms T2DM ACR +ve vs 1,232 ± 36 ms T2DM ACR-ve, *P* = 0.05 ECV: 25.1 ± 2.9 T2DM vs 23.3 ± 3 ms HV, *P* < 0.0.001 ECV: 27.2 ± 4.1 ms T2DM ACR+ve vs 25.1 ± 2.9 ms T2DM ACR-ve, *P* = 0.004
Van den Boomen et al. (68)	Systematic review and Meta-Analysis	831 HTN (739 no LVH HTN)	1101 HV	Native T1	HTN patients (with and without LVH) showed a significant difference between T1 values vs HV (SMD: 0.19; 95% CI 0.01–0.37; *I*2 = 61%; *P* = 0.04) HTN patients without LVH showed no significant difference between the T1 values of HV and HTN patients (SMD: 0.03; 95% CI –0.07–0.13; *I*2 = 2%; *P* = 0.52)
Shang et al. (63)	Cross-sectional prospective	38 T2DM adults	32 HV	Native T1 ECV	Native T1: 1,213.5 ± 57.5 ms T2DM vs 1,212.8 ± 41.4 ms HV, *P* = 0.95 ECV: 30.4 ± 2.9 T2DM vs 27.1 ± 2.4HV, *P* < 0.001 ECV correlated with duration of diabetes (*R* = 0.539, *P* = 0.0005)
Cao et al. (59)	Cross-sectional prospective	50 T2DM patients	50 BMI-matched HV	Native T1 and ECV	ECV: 27.4 ± 2.5% vs. 24.6 ± 2.2%, *p* < 0.001 native T1: 1,026.9 ± 30.0 ms T2DM vs. 1,011.8 ± 26.0 ms HV, *p* = 0.022 Native T1 values correlated with the hemoglobin A1c levels (standardized β = 0.368, *p* = 0.008) ECVs were associated with the HbA1c levels (standardized β = 0.389, *p* = 0.002)
Lam et al. (60)	Cross-sectional prospective	27 T2DM patients	10 HV	Native T1	Native T1: 1,056 ± 31 ms T2DM vs 1,016 ± 21 ms HV, *P* = 0.00051) Native T1 values correlated with the hemoglobin A1c levels (ρ = 0.43, *P* = 0.0088) ECV: 25% ± 0.03 T2DM vs 26% ± 0.02 HV, *P* = 0.47
Gulsin et al. (91)	Cross-sectional prospective	75 T2DM HFpEF adults	65 non-diabetic HFpEF adults	ECV	ECV: 28 ± 5 T2DM HFpEF vs 28 ± 5 non-diabetic HFpEF, *P* < 0.683
Chirinos et al. (90)	Retrospective cross-sectional	32 T2DM HFpEF adults	21 non-diabetic HFpEF adults		ECV: 30.4% T2DM HFpEF vs 27.1% non-diabetic HFpEF, *P* = 0.10
Kucukseymen et al. (62)	Retrospective observational	36 T2DM HFpEF obese adults	45 HV	Native T1	Native T1: 1,129 ± 25 ms T2DM HFpEF vs 1,071 ± 27 ms HV, *P* < 0.001 Native T1: 1,162 ± 37 ms T2DM HFpEF obese vs 1,071 ± 27 ms HV, *P* < 0.0.01
Arcari et al. (65)	Cross-sectional prospective	163 HTN	133 HV		Native T1: 1,102 ± 42 ms HTN vs 1,062 ± 39 ms HV, *P* < 0.001 Discrimination of HTN versus HV: AUC 0.98 (0.93–0.99)
Jiang et al. (78)	Prospective observational	135 T2DM adults	Age-, sex- and BMI-matched 55 HV	Native T1 Pre-contrast T2	Native T1: 1,242.6 ± 230.3 ms T2DM vs 1,209.2 ± 181.7 ms HV, *P* = 0.439 Pre-contrast T2: 41.79 ± 3.41 ms T2DM vs 40.48 ± 2.63 ms HV, *P* = 0.009 ECV: 32.61 ± 4.62 ms vs 27.53 ± 3.05 ms, *P* < 0.001
Bojer et al. (93)	Prospective cross-sectional	264 T2DM adults (207 without LGE, 29 ischemic LGE, 25 non-ischemic LGE, 3 both ischemic and non-ischemic LGE)	25 sex-matched HV	ECV	ECV: 32.2 ± 3.8 T2DM with LGE (ischemic and non-ischemic lesions) vs 28.8 ± 2.7 T2DM without LGE, *P* < 0.0001 ECV: 28.8 ± 2.7 T2DM without LGE vs 26.1 ± 1.5 HV, *P* < 0.0001 ECV: 30.24 ± 3.1 T2DM with non-ischemic LGE vs 28.8 ± 2.7 T2DM without LGE, *P* = 0.01
Khan et al. (88)	Prospective observational	70 T2DM 76 pre-diabetic	296 HV		T2DM was associated with elevated ECV after adjusting for clinical and imaging covariates: β coefficient 1.33 (95% CI, 0.22–2.44); *P* = 0.02 ECV 30% Hazard Ratio for composite events, 3.31 (1.93–5.67), *P* < 0.001
Erden et al. (69)	Observational prospective	83 NAFLD adults	26 HV Liver biopsy for 44 patients		Native T1 MOLLI 3(3)3(3)5: 766.2 (561.2–2,210.2) vs 595.6 (457.6–644.6), *P* < 0.001 Native T1 MOLLI 5(3)3: 656.2 [502.9–1,028.1 vs 564.8 (445.4–605.4)], *P* < 0.001 Native T1 MOLLI 3(2)3(2)5: 744.6 (538.5–2221.5) vs 582.2 (464.0–637.4), *P* < 0.001 Native T1 MOLLI 5(3)3hrc: 638.3 (465.6–931.1) vs 556.8 (442.1–465.6), *P* < 0.001 T2 FLASH: 42.0 (33.2–44.1) NAFLD vs 41.4 (34.0–44.8), *P* = 0.13 T2 TrueFISP: 49.5 (39.4–55.1) NAFLD vs 49.1 (45.1–53.1), *P* = 0.679 Differentiating NAFLD and control group: Native T1 MOLLI 3(3)3(3)5 AUC: 0.976, Accuracy% (95%CI): 94.5 (90.2–98.8), Sensitivity% (95% CI): 92.8 (85.1–96.6), Specificity%: (95% CI) 100 (87.1–100), *P* < 0.001 Differentiating severe steatosis from mild/moderate steatosis Native T1 3(3)3(3)5: AUC: 0.995, Accuracy% (95%CI): 98.7 (96.3–100), Sensitivity% (95% CI): 100 (74.2–100), Specificity% (95% CI): 98.5 (92.1–99.7), *P* < 0.001
Laohabut et al. (92)	Retrospective cohort	188 T2DM adults undergoing CMR for ischemia or viability	551 non-T2DM adults undergoing CMR for ischemia or viability	Native T1 ECV	Native T1: 1,335 ± 75 T2DM vs 1,331 ± 58, *P* = 0.516 ECV: 30.0 ± 5.9 T2DM vs 28.8 ± 4.7, *P* = 0.004 High ECV (HR: 2.01, 95% CI: 1.03–3.93) was identified as independent predictors of cardiovascular events
Idilman et al. (79)	Retrospective observational	23 NAFLD adults (with NASH and without NASH)	Corresponding biopsy	Pre-contrastT2	Pre-contrast T2: 69 ± 7.37 ms NASH vs 61.73 ± 5.99 ms NAFLD without NASH, *p* = 0.016 Pre-contrast T2: 65.44 ± 8.56 NAFLD with lobular inflammation vs NAFLD without lobular inflammation 63.87 ± 5.1 ms, *p* = 0.640 Pre-contrast T2: 68.75 ± 9 NAFLD with portal inflammation vs 64.31 ± 7.3 ms, NAFLD without portal inflammation, *p* = 0.347 Pre-contrast T2 correlated with histology-determined steatosis: *r* = 0.780, *p* < 0.001 Pre-contrast T2 correlated with grade of steatosis: *r* = 0.779, *p* < 0.001 Liver T2 did not correlate with fibrosis stage: *r*_*s*_ = –0.299, *p* = 0.165 Liver T2 correlated with fibrosis stage after adjusting for steatosis: *r* = –0.536, *p* = 0.012 T2 value 65.01 ms discriminated moderate/severe from none/mid steatosis: (area under the curve [AUC] ± SE: 0.875 ± 0.073, 95% confidence interval [CI]: 0.73–1.00, *p* = 0.005), with a sensitivity of 81.3%, specificity of 85.7%, positive predictive value of 85%, and negative predictive value of 82.1%
Salvador et al. (64)	Systematic review and Meta-Analysis	5,053 T2DM		Native T1 ECV	T2DM is associated with a higher degree of MF assessed by ECV% (13 studies; mean difference: 2.09; 95% CI: 0.92–3.27) but not by native T1 (21.74; 95% CI: –1.27 to 44.75).

ACR+ve, albumin: creatinine ratio (indicating persistent micro-albuminuria) positive; ACR-ve, albumin: creatinine ratio (indicating persistent micro-albuminuria) negative; [AUC] ± SE, area under the curve ± standard error; BMI, body mass index; CMR, cardiac MRI; Conc-REMDL, concentric-remodelling; Conc-LVH, concentric left ventricular hypertrophy; cT1, corrected T1; Ecc LVH, eccentric left ventricular hypertrophy; ECV, extra-cellular volume; HbA1c, hemoglobin A1c; HTN, hypertension; HV, healthy volunteers; LGE, late gadolinium enhancement; LVH, left ventricular hypertrophy; MF, myocardial fibrosis; NAFLD, non-alcoholic fatty liver disease; PDFF, proton-density fat fraction; T2DM, type 2 diabetes mellitus.

### 3.1. T_1_ mapping

T_1_ (spin-lattice) relaxation time is the characteristic tissue relaxation constant governing the recovery of longitudinal magnetization (M_z_) back to its thermal equilibrium following a radiofrequency pulse. T_1_ parametric mapping is conventionally achieved by applying magnetization preparation pulses (e.g., Inversion Recovery (IR) or Saturation Recovery (SR) pulses to encode T1 as in MOLLI (1) or in SASHA (57) respectively) preceding the readout to generate the desired T_1_ contrast. The same preparation pulse type is typically applied several times with varying parameter settings, (e.g., inversion delay or saturation delay) to obtain different T_1_ contrast weighted images, which are then used for pixel-wise parametric fitting to the expected signal behavior. The need for several weighted images for parametric fitting (e.g., ∼10 for T1 mapping) and the time required to allow for magnetization recovery reduce the efficiency of the sequence, usually limiting it to one or few 2D slices, especially in the case of cardiac imaging, where the readout is synchronized with the ECG and usually performed at the diastolic cardiac phase.

**FIGURE 3:**
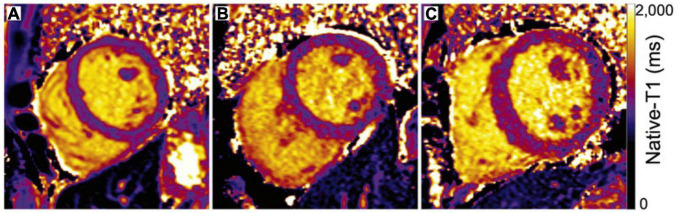
Maps of native-T1 relaxation times in **(A)** a healthy control and **(B)** a diabetic adult with normal left ventricular structural parameters demonstrate similar global mean T1 relaxation times (**A**: 1014 ms, **B**: 1023 ms). **(C)** In contrast, elevated native-T1 relaxation times within the septum, anterior wall, and inferior right ventricular insertion area of a diabetic adult with increased septal wall thickness (1.4 cm) and elevated mass-to-volume ratio (1.4 mg/ml) result in a longer mean left ventricular native-T1 time (**C**: 1,095 ms) compared to either **A** or **B**. Adapted from Lam et al. with permission (60).

Native T_1_ values are prolonged by tissue free water content and are typically shortened by fat and iron. Increased native T_1_ values are seen in oedema and during inflammation (3). Increased T_1_ values are also seen in areas of fibrosis, due to associated expansion of the extracellular space as seen for example in myocardial infarction (MI) and hepatic fibrosis (58). Controversial results about myocardial T_1_ values in diabetic cardiomyopathy have been reported, some studies have concluded significantly increased native T_1_ values in the myocardium of diabetic patients in comparison to controls (59–62) (Figure 3), while other studies have not found a significant difference (20, 63). Meta-analysis of the relevant studies did not show an association of diabetes with native T_1_ time (64). With regards to arterial hypertension, several groups have shown that elevated T_1_ values are found in hypertensive subjects with left ventricular hypertrophy (LVH) compared with those with normal left ventricular myocardial mass and controls (38, 65–67). These results suggest that hypertensive patients have increased myocardial fibrosis, but that this is triggered with the onset of LVH rather than earlier. This could also suggest that interstitial changes in early hypertension (pre-LVH) are non-existent or perhaps are small and not detectable with current applications of T_1_ mapping technique. These findings have been confirmed by a meta-analysis (68). Additionally, in a cohort of patients with NAFLD versus healthy subjects, native liver T_1_ values could differentiate steatotic from non-steatotic livers and showed a strong correlation with history of cardiovascular disease (69).

### 3.2. T_2_ mapping

T_2_ (spin-spin) relaxation time is the MR constant governing the decay of transverse magnetization (M_x,y_) and is dependent on spin-spin interactions. T_2_ parametric mapping is conventionally achieved by applying T2-preparation pulses, with different time durations, before the readout to encode T_2_ (70) and generate the desired T_2_ weighted images. T_2_ mapping requires the acquisition of ∼3–4 T_2_ weighted images including pause heartbeats to allow for magnetization recovery, which collectively reduces the efficiency of the sequence, usually limiting spatial resolution and coverage resulting in the acquisition of only one or a few 2D slices per CMR examination.

T_2_ mapping detects tissue free water content and has been shown very useful for detection of myocardial inflammation and oedema in chronic and acute disease settings (71–76). T_2_ mapping is also used for the differential diagnosis of acute myocardial infarction as it allows detection of the associated oedema and inflammation caused by the acute immune response (77). Jiang et al., demonstrated that diabetes status is related to increased T_2_ values even in asymptomatic individuals, and this is associated with both left ventricular systolic and diastolic function (78). Furthermore, a recent study has demonstrated that there is good correlation between liver T_2_ values and histology determined steatosis (*r* = 0.780, *p* < 0.001) and grade of steatosis (*r* = 0.779, *p* < 0.001). Interestingly, a higher correlation between the liver T_2_ value and percentage of histological steatosis was observed (*r* = 0.838, *p* < 0.001), after adjusting for the fibrosis stage. A T_2_ cut-off value of 65 ms [area under the curve (AUC) ± SE: 0.88 ± 0.07, 95% confidence interval (CI): 0.73–1.00, *p* = 0.005] could discriminate moderate/severe steatosis from none/mild steatosis with a sensitivity of 81%, specificity of 86%, positive predictive value of 85%, and negative predictive value of 82% (79).

### 3.3. T_2_* mapping

T_2_* time captures the dephasing in transverse magnetization (perpendicular to the strong magnetic field) due to the combined effect of field inhomogeneities and susceptibility induced distortions from the magnetised tissue (e.g., high content of paramagnetic materials such as iron) and the spin-spin relaxation related dephasing. The T_2_* relaxation time values are always shorter than or equal to T_2_. Routine evaluation of liver and heart iron content using T_2_* mapping is indicated in patients with suspected iron overload, for instance due to frequent transfusions in thalassaemia and sickle cell patients (80, 81). Increased iron can be co-existing in NAFLD and other chronic liver diseases (82) and emerging evidence suggests that liver iron deposition is associated with worse histopathological features of NASH and disease progression. T_2_* based imaging thus could be used clinically if integrated into clinical guidelines to identify such patients (83, 84). Additionally, iron may interfere with liver T_1_ estimation and thus might contribute to lower accuracy in tissue characterization, if not corrected for.

### 3.4. Extracellular volume

The estimation of the extracellular volume (ECV) is based on the intravenous injection of extracellular gadolinium-based contrast agent (GBCA) with non-protein-bound volume distribution and can be measured using pre- and post-contrast T_1_ mapping (85). The underlying principle is that the T_1_ shortening effect of an extracellular GBCA is directly related to its tissue concentration. The relationship between ECV in the myocardium and blood is approximated by Equation 1, where the change in 1/T_1_ in the tissue and blood pool is used to determine contrast agent concentrations, the ratio of which yields an estimation of ECV, following a correction for red blood cell density in the blood pool (haematocrit, Hct).


ECV⁢myocardium=

(1) $$\frac{\begin{array}{c} \left(\frac{1}{T\,1\,{\text{myo}}_{\text{postGd}}}-\frac{1}{T\,1\,{\text{myo}}_{\text{native}}}\right) \end{array}}{\begin{array}{c} \left(\frac{1}{T\,1\,{\text{blood}}_{\text{postGd}}}-\frac{1}{T\,1\,{\text{blood}}_{\text{native}}}\right) \end{array}}*\left(1-\text{Hct}\right)\,\left(\text{86}\right)$$

**FIGURE 4:**
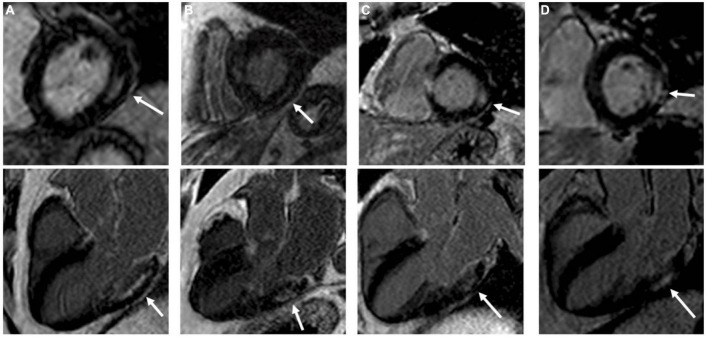
Four type 2 diabetes mellitus patients **(A–D)** with typical non-ischemic late gadolinium enhancement (LGE) lesions with left ventricular short-axis and long-axis images. Non-ischemic lesions are located mid-myocardial, basal and lateral or inferolateral. In segments with non-ischemic LGE lesions, the myocardium remains thick. Adapted from Bojer et al. with permission (93).

**FIGURE 5:**
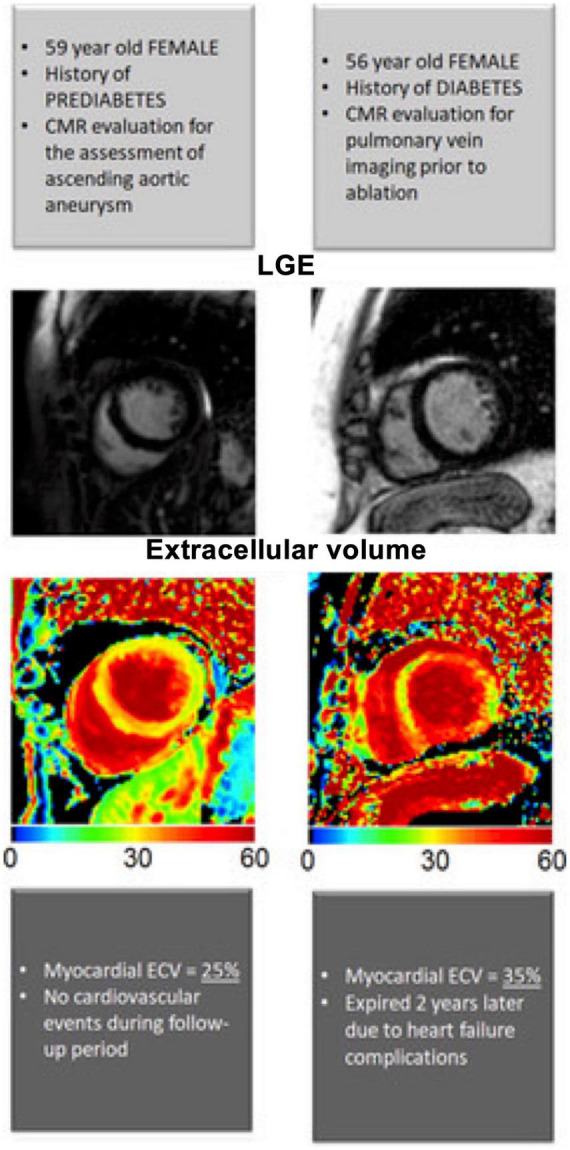
Late gadolinium enhancement images and extracellular volume fraction (ECV) maps by diabetic status. Example of two patients, with prediabetes and diabetes mellitus. Both patients had no late gadolinium enhancement (indicative of replacement fibrosis). The patient with diabetes mellitus had significantly higher amount of ECV (indicative of interstitial fibrosis) compared to the patient without diabetes mellitus. CMR indicates cardiac magnetic resonance. Adapted from Khan et al. with permission (88).

CMR studies have demonstrated, that ECV was significantly higher in HTN LVH subjects versus controls (0.29 ± 0.03 vs. 0.26 ± 0.02, *p* < 0.01) and HTN non-LVH subjects (0.29 ± 0.03 vs. 0.27 ± 0.02, *p* = 0.05) (38, 66). CMR studies showed controversial results with regards to the association of diabetes with increased ECV. Several studies demonstrated that increased ECV is present in diabetic subjects in comparison to controls (20, 59, 61, 63, 78, 87–92). This was found to weakly correlate with hemoglobin A1c levels (59, 89) and the duration of diabetes (63). It was also associated with mortality and/or incident of heart failure admission (87), and constituted an independent risk factor for adverse cardiovascular outcomes (88, 92). It was also associated with late gadolinium enhancement (LGE) lesions that could not be explained by previous infarcts (non-ischemic LGE lesions) and prevalent complications of diabetes (retinopathy, autonomic neuropathy) (93; Figures 4, 5). On the contrary, dissimilar results with regards to the association of diabetes with increased ECV have been suggested by other groups (20, 60, 88, 91, 94, 95). A recently published meta-analysis concluded that diabetes was associated with increased ECV but not with native T_1_ increase and increased ECV was also associated with poor glycaemic control (64).

### 3.5. T1_ρ_ mapping

T1_ρ_ (T1rho) measures the spin-lattice relaxation in the rotating frame, and is a sensitive marker for probing macromolecular water interaction (96). T1_ρ_ has been demonstrated to be sensitive to oedema and fibrotic scar in chronic myocardial infarction. Application of non-contrast T1_ρ_ -mapping in CMR has been reported to discriminate between infarcted and healthy myocardium in animal models (97). Oedema also induces enhancement in T1_ρ_ values, as demonstrated in the area-at-risk in acutely ischemic myocardium, in acute myocarditis and Takotsubo cardiomyopathy (98, 99). This mapping technique sequence is yet to be routinely used in clinical practice. Nevertheless, both oedema and fibrosis are present in the myocardium and liver in NAFLD and future clinical validation in this patient group is warranted to assess its clinical utility as a potential biomarker.

### 3.6. Proton density fat fraction

Proton density fat fraction (PDFF) is a ratio, expressed as a percentage, of the fraction of the MRI-visible protons attributable to fat divided by all MRI-visible protons in that region of the liver attributable to fat and water. Taking advantage of the chemical shift between fat and water, pulse sequences can be used to acquire images at multiple echo times at which fat and water signals have different phases relative to each other (2). MRI-determined PDFF correlates with histologically determined steatosis grade in patients with NAFLD and has been utilized for the assessment of NAFLD in T2DM patients (100, 101) (102). The diagnostic accuracy of MRI-PDFF was further validated by Idilman et al. (103) and Tang et al. (104), both of which demonstrated that MRI-based PDFF assessments correlated closely with histology as assessed by liver biopsy (*r* = 0.82) and explant *ex vivo* histology assessment (*r* = 0.85). Idilman et al. noted that the presence of hepatic fibrosis reduced the correlation between biopsy results and PDFF (103).

## 4. Multiparametric approaches in quantitative MR

Cardiac and liver QMRI, including T_1_, T_2_ and ECV mapping, have emerged as an approach to quantify tissue properties in cardiometabolic disease. Furthermore, in the past years, there has been a growing interest in alternative parameters that may add complementary information. For instance, several studies have shown that T1_ρ_ could be an alternative for the detection of liver (105–107) and myocardium fibrosis (98, 108, 109) without the need of an external contrast agent injection. Nevertheless, at the moment, in clinical practice each quantitative parameter is investigated individually. As a result, sequential, lengthy scans are required to capture multiple parameters in order to accurately describe the various disease phenotypes of cardiometabolic disease (1, 110–112).

Simultaneous multiparametric QMRI, in which the parameters of interest are obtained from a single scan have recently gained attention. An important aspect of this approach is that the parameters should no longer be confounded by each other, promising reliable quantification of the individual parameters in shorter scan time. For instance, liver T_1_ values have been shown to depend strongly on iron content necessitating an additional measurement for liver iron, such as T_2_*mapping, for interpretation of T_1_ values (113). Recent studies in adult and pediatric patients with NAFLD also suggest that hepatic PDFF and T_2_* are strongly correlated with each other *in vivo*. This relationship was observed using different MRI techniques and therefore PDFF and T_2_* value should be considered together when interpreting each of those in human liver (114, 115). Finally, it has been observed that liver fat declines in patients with advanced fibrosis (burnt-out NASH), hence disease progress can be misinterpreted if NAFLD is screened with PDFF for steatosis only (102).

Several models of simultaneous multiparametric QMRI have been investigated in research studies, including methods like joint multiparametric mapping or transient-state imaging approaches (116, 117), magnetic resonance fingerprinting (MRF) (118) and magnetic resonance multitasking (119). Each of them follows a different technical approach, but with the shared goal of providing as many different parametric maps as possible within a single scan. A brief description of each of these and their potential to improve the clinical assessment of cardiometabolic disease is discussed hereafter.

### 4.1. Joint multiparametric mapping

In cardiac MRI, several 2D joint parametric mapping approaches have been proposed. With these approaches the acquisition sequence is generally designed to encode T_1_ and T_2_ simultaneously. Blume et al. (120) (steady-state) and Kvernby et al. (121) (transient state) employed interleaved T_2_-preparation and Inversion Recovery (IR) preparation pulses for T_2_ and T_1_ encoding, respectively. Akçakaya et al. (122) and Guo et al. (123) also used T_2_-preparation for T_2_ encoding but replaced the IR by SR for T_1_ encoding to make the sequence less dependent to heart rate variation. Another approach was proposed by Santini et al. (124); in this case, an IR pulse provides T_1_ encoding, and the subsequent continuous balanced-Steady-State-Free-Precession readout provides the T_2_ encoding.

**FIGURE 6:**
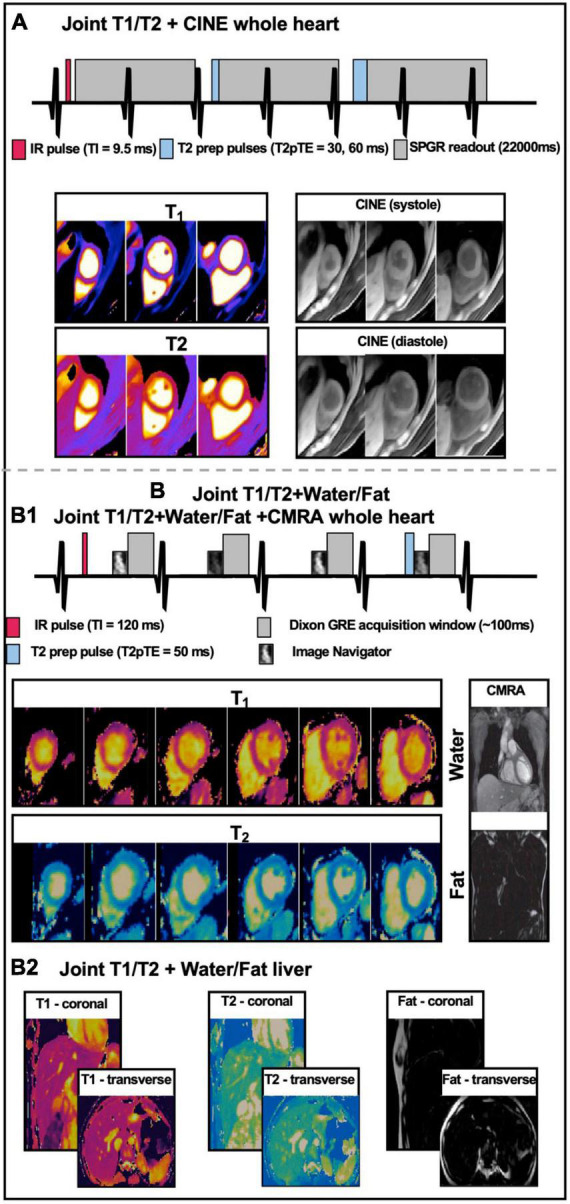
Simplified sequence diagram and corresponding images for three multi-parametric acquisition frameworks. **(A)** Short-axis T_1_ and T_2_ maps, and CINE images at apical, mid-ventricular, and basal levels obtained from a single joint T_1_/T_2_ + CINE free-running whole-heart scan. Figure adapted with permission from Qi et al. (128). **(B1)** Short-axis T_1_ and T_2_ map slices and a representative slice of water and fat CMRA images, obtained from a single joint whole-heart T_1_/T_2_ mapping + Water/FAT CMRA whole heart free-breathing isotropic scan. **(B2)** Representative coronal and transverse slices of joint T_1_, T_2_ maps and Fat images from a 3D isotropic free-breathing liver acquisition.

**FIGURE 7:**
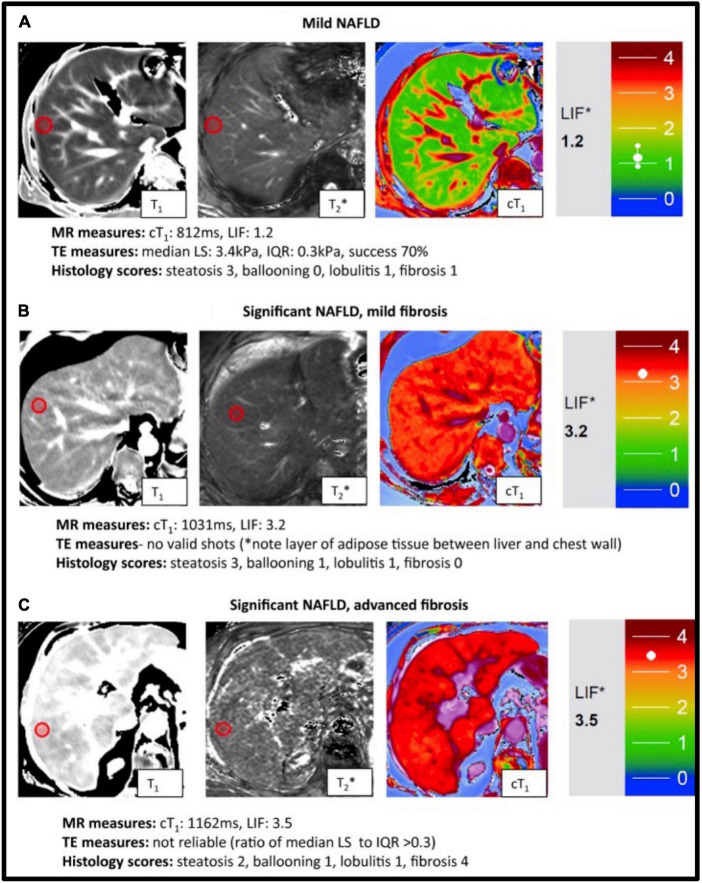
Representative magnetic resonance data with the corresponding transient elastography (TE) and histology data from patients with known or suspected non-alcoholic fatty liver disease NAFLD. T1, T2* mapping values were used to derive the calculated corrected T_1_ maps (cT1) maps and Liver Inflammation and Fibrosis (LIF) scores. Patients were classified based on biopsy findings, using the Fatty Liver Inhibition of Progression (FLIP) algorithm (92), as having: mild disease **(A)**, significant disease/mild fibrosis **(B)** and significant disease/advanced fibrosis **(C)**. Red circles indicate typical regions of interest. There was a significant association between histological fibrosis and MRI LIF scores. Adapted from Pavlides et al. with permission (130).

The multiparametric maps from the aforementioned approaches are obtained after pixel-wise fitting to a sequence-dependent model. However, the need of resting periods for magnetization recovery and the use of breath-holds results in low spatial resolution, limited coverage, and motion artifacts if patients are unable to hold their breath. Applications for cardiac imaging, that sought to address these issues and to enable the acquisition in a clinically-feasible scan time have also been proposed (125) (126; Figure 6A, B1, B2). Those rely on “dictionary matching.” Using this approach, a dictionary is generated which is a compendium of possible signal evolutions for a set of combinations of parameters of interest (such as T_1_ or T_2_), which can be calculated, for example with Bloch simulations (118) or the Extended Phase Graph (127) formalism. The “multi-parametric MR signal” of every pixel is then compared against all entries included in the dictionary by pattern matching (e.g., dot product or least square), to estimate the parameter combination that best represents the measured signal evolution. Dictionaries can also be employed to predict the signal evolution of the transient state; as proposed in MRF. There exist also several examples of multiparametric approaches which were proposed for liver imaging, including water/fat-separated T_1_ mapping (MP-Dixon-GRASP) (128) along with PDFF imaging and water-specific T_1_ mapping [T1(Water)] (PROFIT_1_) (113). An alternative approach has been proposed by Pavlides et al. This includes T_1_ mapping for fibrosis/inflammation imaging and T_2_* mapping for liver iron quantification. The T_1_ measurements of this method are adjusted for the iron level, as high iron levels in the presence of fibrosis can lead to “pseudo-normal” T_1_ values. This was achieved by integrating the results from shortened-MOLLI T_1_ maps and T_2_^^*^ maps in an algorithm that allows to correct for the bias introduced by elevated iron in the T_1_ measurements, yielding iron−corrected T_1_ maps (110, 129). In total, seventy−one patients with suspected NAFLD were recruited within 1 month of liver biopsy and the performance of multiparametric magnetic resonance for the assessment of NASH and fibrosis was evaluated using histology as reference standard (130; Figure 7). Fibrosis stage as analysed on biopsy correlated with MRI-estimated inflammation and fibrosis (*r*_*s*_ = 0.51, *P* < 0.0001). The AUC using this multi-parametric approach for the diagnosis of cirrhosis was 0.85 (95% CI: 0.76–0.95; *P* = 0.0002) and for the diagnosis of mild vs significant NAFLD was 0.89 (95% CI: 0.80–0.98%; *P* < 0.0001). This prospective pilot study demonstrated the potential of multiparametric QMRI to assess the overall disease severity in patients with NAFLD.

### 4.2. Magnetic resonance fingerprinting

**FIGURE 8:**
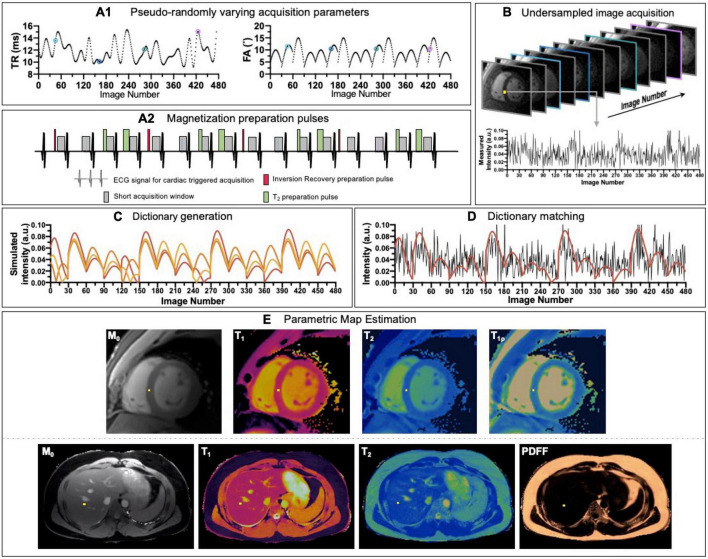
Schematic overview of a cardiac/liver MRF framework. **(A1)** Acquisition parameters such as repetition time (TR) and variable flip angles (FA) may be pseudo-randomly varied throughout acquisition and **(A2)** magnetization preparation pulses are introduced to increment contrast weighing on the desired parameters. **(B)** Highly undersampled images are obtained, and **(C)** a dictionary of different signal evolutions for a range of T_1_/T_2_ (and other parameters of interest) combinations are calculated in parallel. **(D)** Matching the temporal evolution of the signal measured with the dictionary will provide **(E)** inherently co-registered parametric maps of the scanned region.

Most of joint multiparametric approaches presented above are based on steady state imaging and/or discrete sampling of few timepoints along the exponential signal decay, followed by magnetization recovery of the signal and then fit to a certain signal model. There are, however, alternatives like MRF (118) that rely on transient state imaging to generate co-registered multiparametric maps in a single highly efficient scan. In MRF, acquisition parameters such as flip angle and/or repetition time vary pseudo-randomly (Figure 8A1) throughout the scan to generate a unique signal evolution for every tissue, the so-called “fingerprint,” defined by different combination of T_1_, T_2_ and other parameters of interest, when encoded. Parametric encoding can also be increased by interleaving magnetization preparation (e.g., IR or T_2_-preparation) blocks at certain timepoints, similarly to the joint steady-state multiparametric approaches described above (Figure 8A2). In order to obtain a high temporal resolution (i.e., a large number or timepoints in the signal evolution) in an efficient manner, high acceleration factors and thus, highly undersampled images are obtained (Figure 8B). In parallel, a dictionary containing a sufficiently large and representative number of combinations of parameters of interest (e.g., T_1_ or T_2_) is generated using the specific acquisition parameters (Figure 8C).

The “fingerprint” of every voxel is then compared against all the possibilities or entries included in the dictionary by pattern matching to estimate the parameter combination that best explains the measured signal evolution (Figure 8D). In this way, multiparametric co-registered quantitative maps are generated within a single scan (Figure 8E). This dictionary can be reutilized in the subsequent scans provided that the acquisition parameter patterns remain unchanged, which is, however, not the case for cardiac imaging due to subject-specific heart rate variations.

Hamilton et al. (131) proposed for the first time the application of the MRF framework for an ECG-trigged scan for simultaneous T_1_, T_2_ and M_0_ characterization of myocardial tissue. However, given the high flexibility that MRF provides for the extension of the sequence to encode additional parameters, several works have been proposed to extend cardiac MRF to multiparametric assessment, including simultaneous cardiac T_1_/T_2_ maps and PDFF, simultaneous T_1_, T_2_ and T1ρ cardiac MRF and simultaneous T_1_, T_2_, PDFF and T_2_* acquisition (132) (133, 134).

Some of these approaches have been evaluated in healthy subjects (135, 136) and small patient cohorts (137) (138) (139).

For liver imaging, Chen et al. (140) proposed a robust MRF framework where T_1_ and T_2_ 2D maps are obtained on a 3T scanner. This framework has been further extended to include 2D T_1_, T_2_, T_2_* and PDFF mapping in a 14s breath-hold acquisition (141) and initial clinical validation against histological grading from liver biopsies in a cohort of 56 patients with diffuse liver disease has been performed (142). Further advances include evaluating T_1_, T_2_, T_2_*, PDFF and T_1ρ_ mapping (143).

Future clinical validation studies of the aforementioned methods for comprehensive cardiac and liver tissue characterization in cardiometabolic disease are anticipated.

### 4.3. Magnetic resonance multitasking

**FIGURE 9:**
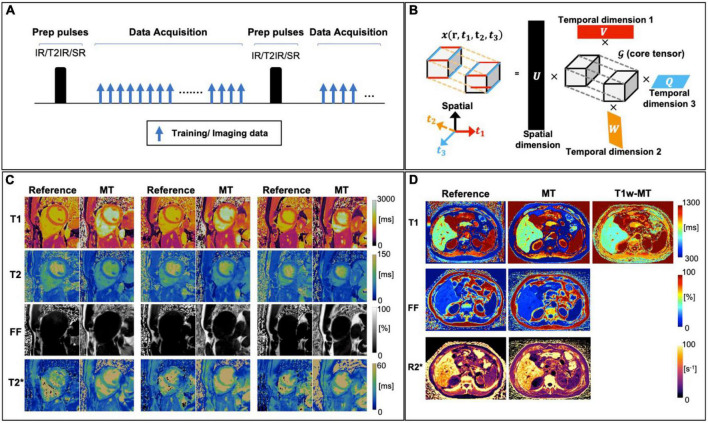
**(A)** A generic sequence diagram for Multitasking technique. The continuous acquisition cycles through different preparation modules (e.g., IR, T_2_-IR, and SR), with FLASH excitations filling the entire recovery period. The training and imaging data are collected in an interleaved way, for resolving temporal and spatial information, respectively. **(B)** An illustration of multi-dimensional image. In this example, the image tensor contains one spatial dimension *r* and three temporal dimensions (can be T1/T2/T2* relaxation, resp motion, cardiac motion, etc.) and its low–rank tensor structure can be explicitly expressed through tensor factorization between 4 sets of basic functions (U, V, W, Q) and a core tensor 𝒢. **(C)** Representative reference and Multitasking cardiac T_1_, T_2_, FF, and T_2_* maps from a healthy volunteer. **(D)** Representative reference and Multitasking liver T1, T1w (water T1), FF, and R2* maps from a patient with non-alcoholic steatohepatitis. Adapted from Cao et al. (59) and Wang et al. (146).

Magnetic resonance multitasking is an alternative approach that enables multiparametric assessment along with the visualization of cardiac and respiratory motion from a single scan. This technique is based, by definition, on a continuous acquisition in which all the possible signal evolutions that are taking place due to different image dynamics (i.e., how the signal would evolve throughout the acquisition due to magnetization relaxation, cardiac or respiratory motion, contrast agent pharmacokinetics or any other cause) are stacked as extra temporal dimension or “tasks” in a high dimensional low rank tensor. In the original work, Christodoulou et al. proposed (119) a cardiac MR multitasking approach where a T_2_-IR prepared free-breathing acquisition leads to simultaneous and motion-resolved T_1_, T_2_ and functional assessment within a single ∼60 s ECG-free scan. At Nyquist sampling rate, the high number of time dimensions considered for this matter would require prohibitive scan times. Christodoulou et al. exploited the low-rank property of the generated tensor, thus the redundant and highly spatio-temporally correlated information is leveraged during the image reconstruction step (Figure 9). Feasibility of the proposed technique has been shown in myocardial T_1_ and ECV mapping (144) and of multi-slice motion-resolved joint T_1_/T_2_ cardiac mapping in a single 3-min free-breathing scan (145). Furthermore, in a recent work, Wang et al. (146) proposed the feasibility of simultaneous 3D quantification of water specific T_1_, PDFF and T_2_^^*^ in a single 5-min scan. Future studies with larger patient cohorts for both heart and liver are warranted for robust clinical validation.

### 4.4. Technical challenges of quantitative MR

Parametric mapping has been widely adopted in clinical practice and constitutes a complementary imaging biomarker in several pathologies. In the theoretical realm, parameters maps depend on the interaction of physics (MRI signal) and the underlying tissue biology. Nevertheless, in clinical practice, several limitations need to be acknowledged, as most mapping techniques depend on several confounding factors. Relaxation time is the result of the combination of the subject, hardware, acquisition, reconstruction algorithm, and map analysis that were used; consequently, all steps in obtaining a relaxation time can add bias or uncertainty to its measurement. A comprehensive review on this scope can be found in Ogier et al. (147). In brief, patients’ heart rate, breathing pattern along with scanner characteristics, such as magnetic field and coils array affect the derived map. With regards to the acquisition and reconstruction techniques, well-established confounding factors include the pulse sequence choice, which is known to affect the quantification of the parameter to be mapped, due to the particular technical and physical limitations of chosen sequence (148). For instance, for T_1_ mapping, different sequences such as MOLLI, shMOLLI, SASHA or SAPPHIRE show different accuracy and precision, as shown by Roujol et al. (149), and dedicated comparative studies have been done to determine which offers better diagnostic power (150). This is also the case for T_2_ mapping, where the use of dedicated T_2_-prep pulses is known to provide significantly underestimated T_2_ values compared to spoiled gradient echo and multi-echo spin echo sequences (151). Prior work has also suggested steady-state preparation schemes to reduce the oscillations that occur in the transient state of steady state free precession due to off-resonance, and the linear flip angle approach was shown to have a superior performance in the presence of large off-resonance frequencies (152). Furthermore, k-space readout, be it linear or centric, has been shown to affect accuracy and precision in T_2_ mapping (148). Similarly, T1rho relaxation is dependent on the applied spin-locking frequency. Additionally, the widely used MOLLI T_1_-mapping sequence is recognized to be confounded by alterations in T_2_, and linear T2prepared balanced steady state free precession values are confounded by T_1_. On some occasions, parameter estimation errors arise when estimating a single parameter without taking into account the effect of other parameters that are inherently coupled; T_2_-prepared sequences will be more prone to T_2_^^*^ susceptibility artifact due to imperfect refocusing of the signal during the preparation whereas T_1_ quantification in the presence of iron will be biased and a corrected T_1_ (cT_1_) is required (130). Other sources of quantification variability such as magnetization transfer (153) or partial volume (154) may affect accuracy and precision. Promisingly, some of these effects can be eliminated or diminished with multi-parametric sequences such as MRF or CMR Multitasking, where several parameters of interest are estimated at the same time for each voxel, removing mis-registration inaccuracies and reducing estimation biases, furthermore multiple corrections can be included on the framework (119, 155–158). Unfortunately, the reproducibility of the aforementioned techniques is still impacted by confounding factors. In particular, multitasking and fingerprinting techniques, where modeling of the signal evolution is utilized to calculate the parameters, error liability is possible where not all influences on signal evolution are included in the model (148) (e.g., the cumulative effect of magnetisation transfer in MOLLI sequence, partial volume, off-resonance effects, magnetisation transfer) (158). Considering those effects on dictionary generation can minimize imperfections. Additionally, the increase of the number of parameters to be estimated for a given number of data points also leads to an increase in the complexity of the acquisition/reconstruction and may affect accuracy and precision as well as increasing computational demands.

### 4.5. Future perspectives for clinical integration of QMRI in cardiometabolic disease

Significant progress has been made to-date to better understand the histological alterations of cardiac and hepatic tissue in cardiometabolic disease and their potential correlation to QMRI techniques. The quantification of cardiac fibrosis in T2DM has been extensively studied with T1 mapping and ECV methods and this has been associated with adverse cardiovascular events. Several pilot studies have also demonstrated myocardial fibrosis in hypertension. Parametric tissue characterization has demonstrated hepatic fibrosis, steatosis and inflammation in proof-of-principle studies in NAFLD. Nevertheless, the scope of QMRI in cardiometabolic disease has not been fully investigated. This is attributed primarily to two factors. Firstly, the standardization of the existing clinical single-parametric mapping techniques has been suboptimal and current guidelines suggest the generation of site-specific normal ranges. Validation and subsequent standardization of the new methods has not been performed either and is a crucial step to enhance clinical uptake. Furthermore, the reproducibility and robustness of the proposed methods needs to be ensured in multi-center and multi-vendor studies. The design of prospective, longitudinal studies tailored to the relevant clinical questions, incorporating the novel technologies available, is also mandatory to expedite clinical adoption (159). Efforts toward reproducibility and standardization can often be accelerated through an overarching international organization that many parties trust, such as the Quantitative Image Biomarker Alliance of the Radiological Society of North America and the Quantitative MR Study of the International Society for Magnetic Resonance in Medicine (ISMRM).

Additionally, advanced acquisition schemes often come at the cost of lengthy acquisition and post-processing times. Further applications of multi-parametric QMRI that incorporate deep-learning based approaches demonstrate promising results at no extra time-cost either at acquisition or image processing level and would augment the diagnostic information (160–162). This could also allow the exploration of additional contrast weightings, including for example tissue diffusion. Furthermore, in view of the multi-organ manifestations of cardiometabolic disease, studies investigating simultaneously the liver and cardiac tissue are anticipated, to gain insight into the pathophysiology of cardiac-liver axis (140).

## 5. Limitations

Ongoing research in cardiometabolic disease has discovered novel mechanistic pathways across various organ systems, including cardiac and skeletal muscle, pancreas, liver, adipose tissue and microcirculation. An elaborate review on inter-organ pathogenetic interrogation and multimodality imaging perspective is out of scope of this article and has been covered elsewhere (4). Additional MRI techniques that have been applied in cardiometabolic disease include magnetic resonance elastography and magnetic resonance spectroscopy. Magnetic resonance elastography has been primarily utilized for the evaluation of liver stiffness. It relies on the demonstration of propagating shear waves within the liver employing a phase-contrast type sequence (163). Magnetic Resonance spectroscopy investigates cardiac and hepatic metabolism *in vivo* by measuring proton signals as a function of their resonance frequency. By using the gyromagnetic properties of ^1^H, ^31^P, ^13^C, and ^23^Na, Magnetic Resonance Spectroscopy relates energy metabolism to (dys)function of the heart (164, 165). This article, which focuses on relaxation and proton-density fat fraction mapping techniques, cannot elaborate on the aforementioned methods due to space constraints. The reader is directed to (163–165) for deeper insights into the physics and applications of the respective methodology.

## 6. Conclusion

Cardiometabolic disease is a cluster of complex diseases that involve changes in the physiology of myocardial and hepatic tissue. Quantitative MR imaging is a valuable tool to characterize this disease, although a single quantitative parameter may not provide sufficient information. Simultaneous multiparametric MRI has demonstrated the feasibility of obtaining fast, co-registered multiple parametric maps within a single short MR scan and is promising for comprehensive understanding of the disease. QMRI frameworks are currently at a transition point between development and clinical adoption. Inclusion of standardization agreements, quality control protocols, and reproducibility assessment are essential for the clinical validation and uptake of these new promising techniques to gain further insight into cardiometabolic disease.

## Author contributions

AF, CV, and CP devised and wrote the manuscript. CP and RB reviewed the manuscript. All authors contributed to the article and approved the submitted version.
